# Parametric imaging of myocardial viability using ^15^O-labelled water and PET/CT: comparison with late gadolinium-enhanced CMR

**DOI:** 10.1007/s00259-012-2134-8

**Published:** 2012-05-11

**Authors:** Stefan de Haan, Hendrik J. Harms, Mark Lubberink, Cornelis P. Allaart, Ibrahim Danad, Weena J. Y. Chen, Michaela Diamant, Albert C. van Rossum, Hidehiro Iida, Adriaan A. Lammertsma, Paul Knaapen

**Affiliations:** 1 Department of Cardiology, VU University Medical Center, De Boelelaan 1118, 1081 HV Amsterdam, The Netherlands; 2 Department of Nuclear Medicine & PET Research, VU University Medical Center, Amsterdam, The Netherlands; 3 Department of Internal Medicine, Diabetes Center, Amsterdam, The Netherlands; 4 Institute for Cardiovascular Research (ICaR-VU), VU University Medical Center, Amsterdam, The Netherlands; 5 Department of Investigative Radiology, National Cerebral and Cardiovascular Center Research Institute, Osaka, Japan

**Keywords:** PET/CT, CMR, Perfusable tissue index, Late gadolinium enhancement

## Abstract

**Purpose:**

The perfusable tissue index (PTI) is a marker of myocardial viability. Recent technological advances have made it possible to generate parametric PTI images from a single [^15^O]H_2_O PET/CT scan. The purpose of this study was to validate these parametric PTI images.

**Methods:**

The study population comprised 46 patients with documented or suspected coronary artery disease who were studied with [^15^O]H_2_O PET and late gadolinium-enhanced (LGE) cardiac magnetic resonance imaging (CMR).

**Results:**

Of the 736 myocardial segments included, 364 showed some degree of LGE. PTI and perfusable tissue fraction (PTF) diminished with increasing LGE. The areas under the curve of the PTI and PTF, used to predict (near) transmural LGE on CMR, were 0.86 and 0.87, respectively. Optimal sensitivity and specificity were 91 % and 73 % for PTI and 69 % and 87 % for PTF, respectively.

**Conclusion:**

PTI and PTF assessed with a single [^15^O]H_2_O scan can be utilized as markers of myocardial viability in patients with coronary artery disease.

## Introduction

Assessment of myocardial viability in patients with coronary artery disease is of great clinical importance, as dysfunctional but viable myocardium has the ability to regain contractility after coronary revascularization with subsequent improvements in cardiac function and prognosis [1]. The perfusable tissue index (PTI) can be used as a marker of myocardial viability, having been validated in patients with ischaemic heart disease [2–7]. However, PTI has never been used in clinical practice, primarily due to the complex imaging protocol consisting of dynamic [^15^O]H_2_O and [^15^O]CO scans, together with a transmission scan, and the lack of high-quality, clinically usable parametric images [6]. Recently, however, a method was developed to derive the PTI from a single [^15^O]H_2_O PET/CT scan [8]. Given the current potential of clinical [^15^O]H_2_O-based perfusion imaging and the rapid growth in the availability of cardiac PET/CT systems, PTI viability measurements could become incorporated into clinical practice [9, 10]. Parametric PTI images derived from [^15^O]H_2_O PET/CT scans, however, have not yet been validated. The purpose of this study was to compare these novel parametric PTI images with late gadolinium-enhanced cardiovascular magnetic resonance (LGE-CMR) imaging, an established method for quantifying scar size and a marker of viability.

## Materials and methods

### Study population

The study population comprised 46 patients with documented or suspected coronary artery disease who had been studied using both PET/CT and CMR within a 2-month period. All patients were in a stable clinical condition and no ischaemic events or revascularizations had occurred during the period between the two examinations. Patients with contraindications for PET/CT or CMR (e.g. pacemaker, claustrophobia, atrial fibrillation) were excluded. The study was approved by the institutional Medical Ethics Review Committee, and all participants gave written informed consent prior to inclusion.

### PET/CT image acquisition

[^15^O]H_2_O scans were acquired using a Gemini TF-64 (Philips Healthcare, Best, The Netherlands) PET/CT scanner. [^15^O]H_2_O (370 MBq) was injected as a 5-ml bolus (0.8 ml s^−1^) followed by a 35-ml saline flush (2 ml s^−1^), simultaneously starting a 6-min list mode emission scan. This scan was followed immediately by a respiration-averaged slow low-dose (LD) CT scan (55 mAs, rotation time 1.5 s, pitch 0.825, collimation 16 × 0.625, acquiring 20 cm in 37 s) during normal breathing. All scans were checked for misalignment between the LD CT scan and the [^15^O]H_2_O scan; in none of the patients were corrections needed. Dynamic [^15^O]H_2_O images were reconstructed into 22 frames (1 × 10, 8 × 5, 4 × 10, 2 × 15, 3 × 20, 2 × 30, 2 × 60 s) using the three-dimensional row action maximum likelihood algorithm and applying all appropriate corrections for the scanner, normalization, dead time, decay, scatter, randoms and attenuation based on the corresponding LD CT scan.

Using CAPP software (Siemens/CTI, Knoxville, TN), regions of interest (ROIs) of 1 cm diameter were placed over the ascending aorta in at least ten transaxial image planes of the frame showing the first pass of the injected bolus. These ROIs were combined into one volume of interest (VOI) for the ascending aorta. A second set of ROIs were placed over the right ventricle (RV) cavity in at least five transaxial planes, with ROI boundaries at least 1 cm from the RV wall to avoid spill-in from myocardial activity. Again, these ROIs were combined into one VOI for the RV. Both VOIs were then projected onto all dynamic [^15^O]H_2_O images, thereby generating arterial (C_A_(t)) and RV (C_RV_(t)) time–activity curves.

### PET/CT image analysis

**Fig. 1:**
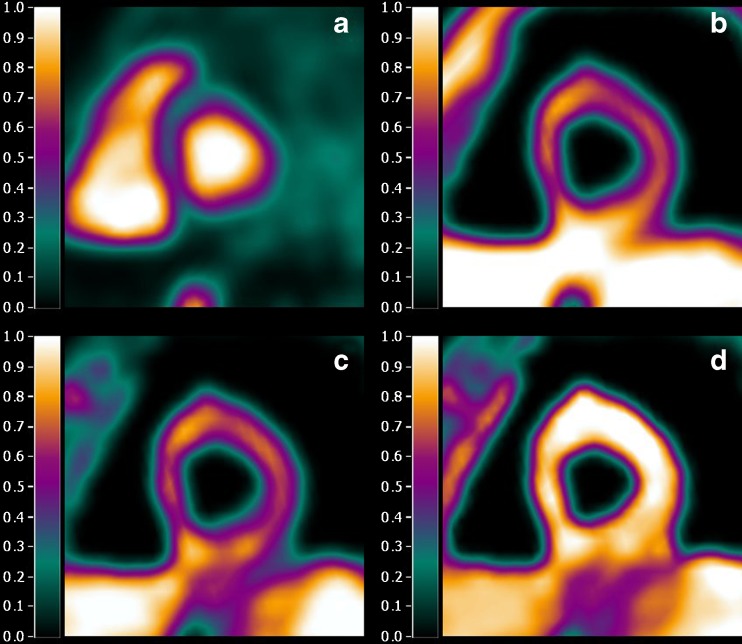
Example of short axis images of (**a**) blood volume, (**b**) ATF (g ml^−1^), (**c**) PTF (g ml^−1^), and (**d**) PTI in a patient without coronary artery disease

Parametric PTI images were calculated as previously described [8]. In brief, parametric images of myocardial blood flow (MBF), perfusable tissue fraction (PTF), and arterial and venous blood volume fractions were calculated using a basis function implementation of the standard single-tissue compartment model for [^15^O]H_2_O [11–14]. Parametric images of arterial and venous blood volume fractions were subtracted from normalized CT transmission images, resulting in parametric anatomic tissue fraction (ATF) images. Finally, parametric PTI images were calculated as the ratio of PTF and ATF images (Fig. 1). All parametric images were generated using software developed in-house. Finally, 16 myocardial VOIs were defined manually on parametric PTF images, according to the 16 segments model of the American Heart Association [16], after which this VOI template was projected onto the parametric PTI images.

### CMR image acquisition

CMR studies were performed on a 1.5-T whole-body scanner (Magnetom Sonata or Avanto; Siemens, Erlangen, Germany), using a six-channel phased-array body coil. After survey scans, a retrotriggered, balanced steady-state free precession gradient-echo sequence was used for cine imaging. Image parameters included: slice thickness 5 mm, slice gap 5 mm, temporal resolution <50 ms, repetition time 3.2 ms, echo time 1.54 ms, flip angle 60°, typical image resolution 1.3 × 1.6 × 5.0 mm. The cardiac cycle consisted of 20 phases. After obtaining four-, three- and two-chamber view cines, stacks of 10 to 12 short-axis slices were acquired to fully cover the left ventricle (LV). Cine images were acquired during breath-hold with mild expiration. Contrast images were acquired 10 to 15 min after administration of 0.2 mmol kg^−1^ gadolinium-DTPA in the same views as in the cine images, using a two-dimensional segmented inversion-recovery prepared gradient echo sequence (TE 4.4 ms, TR 9.8 ms, inversion time 250 to 300 ms, typical voxel size 1.3 × 1.6 × 5.0 mm).

### CMR image analysis

Images were analysed off-line using the software package MASS (MR Analytical Software System; Medis, Leiden, The Netherlands). First, short-axis cine images were analysed. The endocardial and epicardial borders of the LV were outlined manually in both end-diastolic and end-systolic phases of all short-axis images. Papillary muscles were included in the LV volume. End-diastolic volume, end-systolic volume and ejection fraction were computed using these analyses. Subsequently, the endocardial and epicardial contours of the LGE images were traced manually. The amount of fibrosis was calculated using the full-width at half-maximum method, which defines fibrosis as myocardial tissue with a signal intensity ≥50 % of the maximum hyperenhancement intensity. If no enhancement was found in a slice, the maximum signal of the nearest slice with enhancement was used. If two neighbouring slices showed enhancement, maximum signals were averaged. All areas of enhancement were quantified by computer-assisted planimetry on each of the short-axis images and the segmental extent of enhancement was expressed as a percentage of the segmental area. CMR images were analysed according to the same 16-segment model as used for the parametric PET images. Finally, myocardial segments were graded as viable or nonviable using the previously defined cut-off value of 50 % LGE per segment [15, 17].

### Statistical analysis

Continuous variables are presented as means ± SD, and categorical data are summarized as frequencies and percentages. Multiple datasets were compared using analysis of variance (ANOVA), and specific differences were identified using Student’s *t*-test with Bonferroni inequality adjustment. Receiver operating characteristic curves were generated for PTF, PTI and MBF for the prediction of myocardial viability assessed by LGE CMR. The area under the curve (AUC) was considered a measure of accuracy to discriminate between viable and nonviable myocardium. A *p* value of <0.05 was considered statistically significant.

## Results

### Baseline characteristics

Baseline characteristics of the patient population are shown in Table 1. LGE was seen in the CMR images of 34 patients (74 %). Of the 736 myocardial segments, 364 (49 %) showed some degree of LGE.

**Table 1:** Patient characteristics (*n* = 46)

Characteristic	Value
Age (years)	65 ± 10
Gender (male)	36 (78 %)
Previous myocardial infarction	34 (74 %)
LV end-diastolic volume (ml)	226 ± 65
LV end-systolic volume (ml)	135 ± 71
LV ejection fraction (%)	42 ± 16
LV mass (g)	132 ± 39

### PET/CT and CMR parameters

PET/CT and CMR data are summarized in Table 2. There was a gradual decrease in PTF, PTI and MBF values with increasing degree of LGE on CMR images (*p* < 0.001 by ANOVA). ATF values remained relatively constant, except for a significant decrease in the (near) transmurally enhanced segments (*p* < 0.001 by ANOVA).

**Table 2:** Segmental PET/CT and LGE data

	Extent of LGE (%)					
	Control (*n* = 372)	0–25 (*n* = 190)	25–50 (*n* = 83)	50–75 (*n* = 54)	≥75 (*n* = 37)	*p* value (ANOVA)
ATF	0.76 ± 0.09	0.77 ± 0.09	0.75 ± 0.11	0.73 ± 0.08	0.68 ± 0.11**	<0.001
PTF (g ml^−1^)	0.72 ± 0.08	0.72 ± 0.09	0.70 ± 0.09	0.60 ± 0.08**	0.51 ± 0.12***	<0.001
PTI	0.91 ± 0.08	0.89 ± 0.09	0.89 ± 0.09	0.77 ± 0.10**	0.70 ± 0.16***	<0.001
MBF (ml g^−1^ min^−1^)	1.02 ± 0.30	0.91 ± 0.26*	0.85 ± 0.30*	0.83 ± 0.24*	0.67 ± 0.30**	<0.001

*p < 0.05 vs. control; **p < 0.05 vs. control, 0–25 % LGE and 25–50 % LGE; ***p < 0.05 vs. control, 0–25 % LGE, 25–50 % LGE and 50–75 % LGE.

### Predictive values for viability

**Fig. 2:**
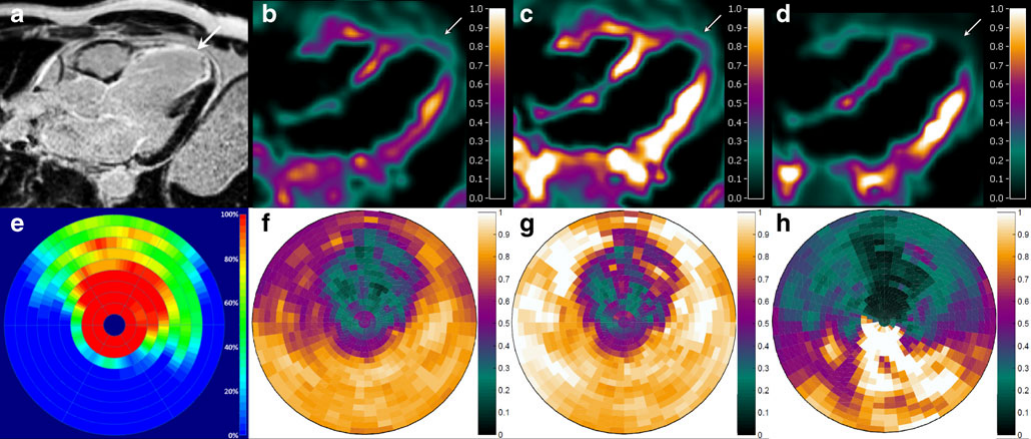
Long axis images (**a**–**d**) and polar maps (**e**–**h**) in a patient with an anterior myocardial infarction: **a**, **e** CMR with LGE (*arrow*, % transmurality); **b**, **f** PTF (g ml^−1^); **c**, **g** PTI; **d**, **h** MBF (ml g^−1^ min^−1^)

Figure 2 shows a concordant pattern between parametric PTF, PTI and LGE CMR images in a patient with ischaemic cardiomyopathy after an anterior myocardial infarction.

**Fig. 3:**
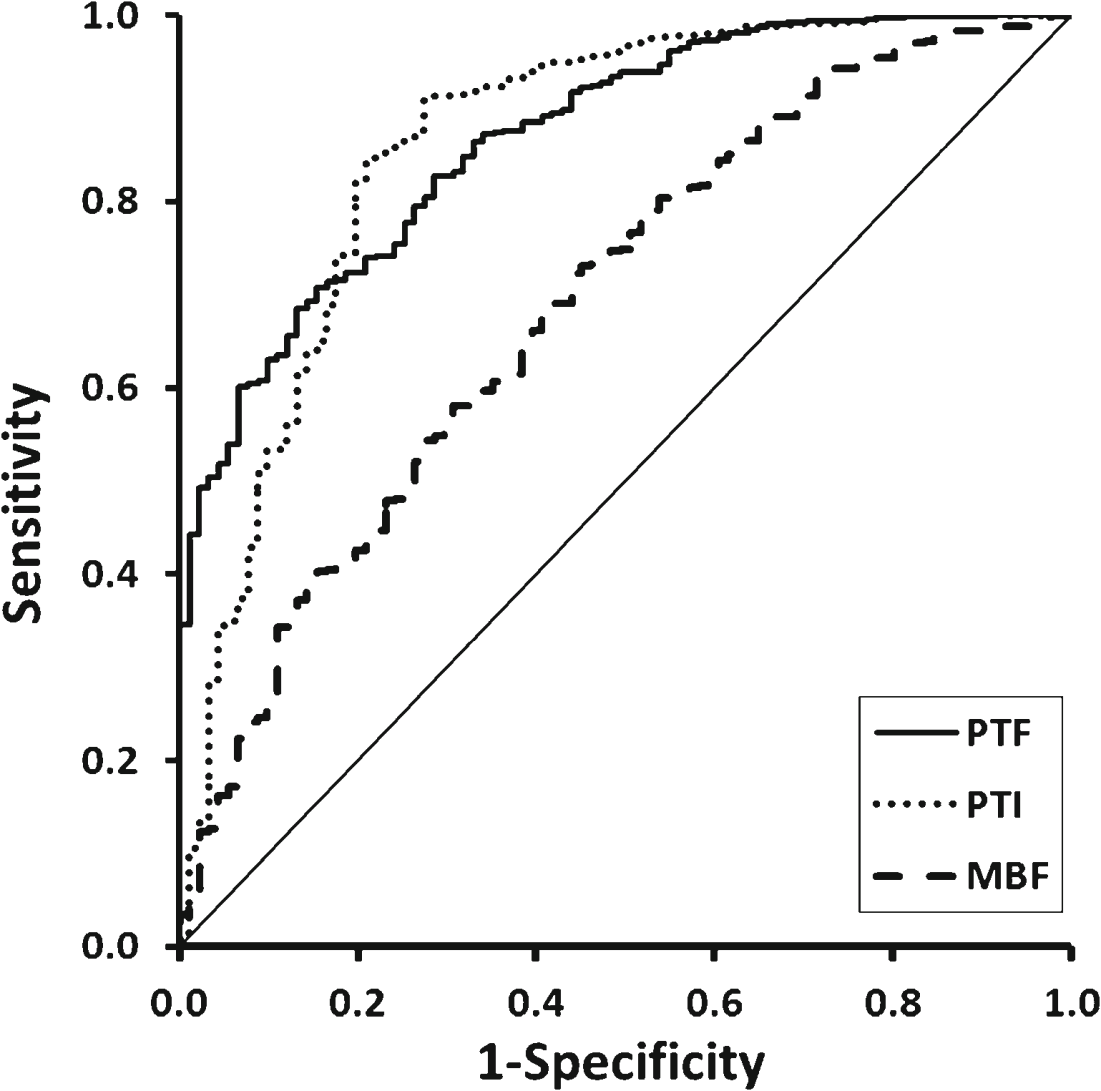
Long axis images in a patient with a nontransmural anterior myocardial infarction: **a** CMR with LGE; **b** PTI

**Fig. 4:**
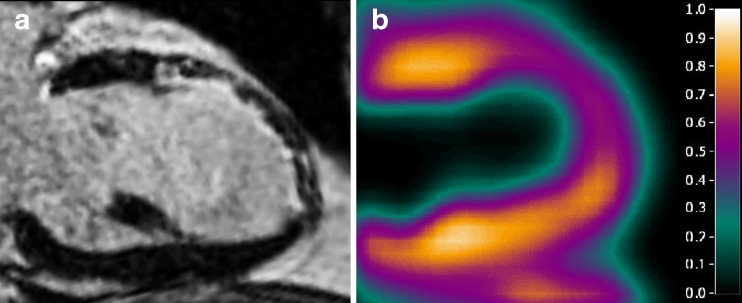
Receiver operator characteristics curves for the abilities of PTF, PTI and MBF to differentiate between viable and nonviable segments based on late gadolinium enhancement

Using CMR as a reference, 91 of 364 (25 %) segments showing some degree of LGE were judged to be nonviable (LGE >50 %). As shown in Fig. 4, the values of PTF and PTI for predicting myocardial viability in all 736 segments were comparable (AUC 0.87, CI 0.83–0.90, and 0.86, CI 0.82–0.91, respectively, *p* = 0.541). MBF was able to predict myocardial viability with less accurate (AUC 0.69, CI 0.63–0.75, *p* < 0.001). Optimal cut-off values of PTF, PTI and MBF for predicting (near) transmural LGE on CMR were 0.69 g ml^−1^, 0.80, and 0.78 ml min^−1^ g^−1^ with sensitivities of 69 %, 91 % and 72 %, and specificities of 87 %, 73 % and 56 %, respectively. Figure 3 shows an example of a patient with a nontransmural scar and a corresponding relatively high PTI.

## Discussion

The present study was conducted to validate the use of a parametric myocardial viability imaging technique using [^15^O]H_2_O PET/CT in patients with ischaemic heart disease. Viability assessed using parametric PTF and PTI imaging was in good agreement with that assessed using LGE CMR. Furthermore, these images and myocardial perfusion imaging are obtained simultaneously allowing both myocardial viability and ischaemia to be evaluated in a single scanning session.

[^15^O]H_2_O is generally considered to be the gold standard for absolute quantification of myocardial perfusion in vivo [10, 18, 19]. Apart from MBF, [^15^O]H_2_O also provides estimates of the extent of myocardium within a ROI that is able to exchange water rapidly, i.e. PTF. Subsequently, PTF can be corrected for partial volume effects by dividing it by its anatomical counterpart ATF, resulting in PTI [5]. It has been shown that both PTF and PTI can identify myocardial scarring and thus can act as markers of viability [6]. Only recently, however, a method has been developed that enables generation parametric ATF, PTF and PTI images from a single [^15^O]H_2_O PET/CT scan [8]. This obviates the need for a separate [^15^O]CO blood volume scan. In addition, the traditional (long) transmission scan can be replaced by a (rapid) low-dose CT scan, thereby shortening the total scanning time substantially. Now that the method has been shown to produce high-quality parametric images, its implementation into clinical practice needs investigation.

Using the described parametric imaging approach, the present study demonstrated that ATF was relatively constant independent of tissue characteristics of the myocardium. Only in (near) transmurally infarcted segments was ATF significantly reduced, most likely due to wall thinning, as ATF is prone to partial volume effects. In contrast, PTF and PTI progressively decreased with increasing extent of scarring, as shown by LGE CMR. These results are in line with those of previous studies in which reductions in both PTF and PTI were observed in nonviable scarred myocardium [6]. PTI in control segments appeared to be somewhat lower than the expected value of unity for normal segments (0.91 ± 0.08). This phenomenon has previously been observed in cardiomyopathy in both animal experiments and human studies [6, 20]. Slight misalignment between PET and CMR segments may have occurred to additionally account for this reduction in PTI. Furthermore, the presence of interstitial fibrosis in dilated cardiomyopathy may also explain this reduction in ‘normal’ segments that remains undetected on LGE CMR [21, 22].

Taking LGE as a reference, the optimal cut-off values for discriminating between viable and nonviable myocardium were 0.69 g ml^−1^ and 0.80 for PTF and PTI, respectively. AUC analysis revealed that the diagnostic accuracies with the two PET parameters were comparable. This suggests that the use of parametric PTF alone may suffice to assess viability. Although this would reduce the data processing time, it would not affect the scanning protocol, as for both parameters a dynamic [^15^O]H_2_O PET scan in combination with a low-dose CT scan are required. In addition, the sensitivity of PTI exceeded that of PTF (91 % and 68 %, respectively), whereas for specificity the opposite pattern was observed (73 % and 78 %, respectively). Although further study is need to determine the cause of this discrepancy, as a potential clinical marker of viability PTI may be favoured over PTF to reduce false-negative findings in patients who might benefit from revascularization. It is of interest to note that, compared to PTF and PTI, MBF performed relatively poorly in distinguishing viable from nonviable tissue, rendering it less suitable for viability imaging. Previous studies have indicated that the optimal cut-off value for PTI is in the range 0.7–0.9. The threshold observed in the present study corresponds with that range.

LGE was used as a surrogate end-point of myocardial viability instead of functional recovery of dysfunctional myocardium after revascularization. LGE has been shown to be a good, but not perfect, marker of myocardial viability [15, 17]. Therefore, the results should be interpreted with caution, and more studies are warranted to establish the value of parametric PTI as a viability marker.

### Conclusion

PTF and PTI, obtained from a single [^15^O]H_2_O PET/CT scan, can be used as markers of myocardial viability in patients with coronary artery disease.
